# Perceptions of Clinical Experience and Scientific Evidence in Medical Decision Making: A Survey of a Stratified Random Sample of Swedish Health Care Professionals

**DOI:** 10.1177/0272989X241234318

**Published:** 2024-03-16

**Authors:** Barry Dewitt, Johannes Persson, Annika Wallin

**Affiliations:** Department of Engineering and Public Policy, Carnegie Mellon University, Pittsburgh, PA, USA; Department of Philosophy, Lund University, Lund, Sweden; Department of Cognitive Science, Lund University, Lund, Sweden

**Keywords:** clinical experience, evidence-based medicine, epistemology, medical education, health policy

## Abstract

**Background:**

Evidence-based medicine recognizes that clinical expertise gained through experience is essential to good medical practice. However, it is not known what beliefs clinicians hold about how personal clinical experience and scientific knowledge contribute to their clinical decision making and how those beliefs vary between professions, which themselves vary along relevant characteristics, such as their evidence base.

**Design:**

We investigate how years in the profession influence health care professionals’ beliefs about science and their clinical experience through surveys administered to random samples of Swedish physicians, nurses, occupational therapists, dentists, and dental hygienists. The sampling frame was each profession’s most recent occupational registry.

**Results:**

Participants (*N* = 1,627, 46% response rate) viewed science as more important for decision making, more certain, and more systematic than experience. Differences among the professions were greatest for systematicity, where physicians saw the largest gap between the 2 types of knowledge across all levels of professional experience. The effect of years in the profession varied; it had little effect on assessments of importance across all professions but otherwise tended to decrease the difference between assessments of science and experience. Physicians placed the greatest emphasis on science over clinical experience among the 5 professions surveyed.

**Conclusions:**

Health care professions appear to share some attitudes toward professional knowledge, despite the variation in the age of the professions and the scientific knowledge base available to practitioners. Training and policy making about clinical decision making might improve by accounting for the ways in which knowledge is understood across the professions.

**Highlights:**

- Study participants, representing 5 health care professions—medicine, nursing, occupational therapy, dentistry, and dental hygiene—viewed science as more important for decision making, more certain, and more systematic than their personal clinical experience.
- Of all the professions represented in the study, physicians saw the greatest differences between the 2 types of knowledge.
- The effect of years of professional experience varied but tended to be small, attenuating the differences seen between science and clinical experience.

## Introduction

### Scientific Background and Rationale

Professional experience involves the accumulation of skills and practical knowledge. Once a profession’s standardized training is complete, professional experience increases through practice of the profession, which usually includes much less structured education than the formal training period. Evidence-based medicine recognizes that clinical expertise gained through experience is essential to good medical practice.^1^ Although health care professionals do their best to keep up to date with the latest scientific advancements in their fields, limited time for in-service training means that they eventually know less about the latest developments than their more recently trained colleagues do.^2^ Surveys indicate that physicians and nurses think updating knowledge and skills are important professional standards, although competency assessments are rare after licensure.^3,4^ Evidence is mixed regarding whether recency of formal training matters more to patient outcomes than experience does. There is evidence that patients of hospitalists with 1 y of experience or less have higher 30-d and hospital mortality rates than do patients of hospitalists with more than 2 y of experience,^5^ as well as evidence that patients of hospitalists younger than 40 y have a lower 30-d mortality rate than patients of hospitalists 40 y and older^6,7^—with the exception of hospitalists treating high volumes (whose patients had the lowest mortality rates, cf. ref. 6). In contrast, patients of younger surgeons have higher 30-d and hospital mortality than patients of older surgeons do.^8^

The relationship between the scientific knowledge learned during training and experience-based knowledge—some gained in training, most gained afterward—is complex. On one hand, scientific evidence has the advantage of being systematically produced and tested; on the other hand, clinical experience is directly related to the context in which it is used. Two important dimensions on which scientific evidence and personal experience may differ are their certainty and systematicity. Personal experience is tied to the specific situations in which it was produced and personal knowledge based on these situations; thus, it includes features of which one might be certain and features of which one might be less so. Scientific knowledge will (ideally) be based on a larger number of observations. On the other hand, it often comes at the cost of being removed from the specifics of a given clinical encounter. Scientific evidence will therefore be more or less certain in relation to applicability. The production of scientific knowledge will also follow established procedures that are neither possible nor desirable in everyday health care (e.g., randomized controlled trials). It will also relate to preconceived theory in an orderly manner to an extent that personal knowledge, due to the uncontrolled way in which it is generated, will not. Therefore, the two will vary also with respect to how systematic they are in how knowledge is generated, described, and substantiated.^9,10^ (The Swedish word “systematisk” means precisely this).

It is even possible that some areas of health care are further removed from the possibility to follow gold standards, because of “mere” practical concerns. For instance, the differences between surgeons and hospitalists described above may suggest that surgery is more dependent on manual skills, which to a high extent depend on experience.^11^ Familiar claims that clinical equipoise is so hard to attain in surgery that randomized controlled trials are hampered^12^ reinforce that view. In a similar vein, some health professions may have better means to follow patients over time (e.g., occupational therapists [OTs] working with a particular person all through recovery) than others (e.g., staff in emergency rooms).

Health care professionals are expected to stay current in their respective fields. “Current” refers to a breadth of knowledge: new professional guidelines, new biomedical and social science studies in the literature, and new “best practices.” In Sweden, the parliament recently demanded that health care personnel are required to and given the right to continuing education,^13^ but this has yet to be implemented. In an opinion piece written by representatives from Sweden’s health care unions, it is claimed that actual continuing education for physicians has almost halved since 2005, to 5 d per year in 2019, with other health care professions receiving even less.^14^

Here, we use a survey to examine epistemological beliefs about the contributions of science and experience in day-to-day health care. A random sample of Swedish health care professionals—doctors, nurses, OTs, dentists, and dental hygienists—rated how important science and experience each are for medical decision making as well as how certain and systematic they perceive those types of knowledge to be (see the Appendix for the full survey). That knowledge can differ in its importance, in how orderly it has been produced (its systematicity), and in how trustworthy it appears to be (its certainty) are uncontroversial and common ideas in Swedish, and we fully expected our participants to interpret them coherently.

In the Nordic countries, a third concept related to both scientific evidence and personal experience exists. This is “beprövad erfarenhet,” literally *proven experience*. The concept is present as early as decrees from the late 19th century^15^ and is part of the Swedish Patient Act^16^ and Patient Safety Act,^17^ both directed at health care. Interestingly, the concept has no formal definition, but health care personnel interpret it as referring to, among other things, that interventions and methods are tested (in practice) and that they are part of an accepted practice^18^ (cf. Refs. 19–21). As proven experience is part of Swedish health care personnels’ ideas on evidence, participants were also asked to respond to items on this concept. Including the concept of proven experience is thus likely to shift participants’ perception of personal experience to their individual experience, rather than to what is “commonly known” in their health care context (which would instead be categorized as proven experience).

Regardless of profession, we expect more experienced health care professionals to perceive a smaller difference between science and their personal experience in terms of how important, certain, and systematic they are than less experienced members of the same profession. This is because a larger amount of personal experience provides the practitioner with more data, which allows for more certain conclusions than if the experience base had been smaller. It will likely also produce a variation of circumstances that allows for some systematicity in learning from experience. In addition, more certain and systematic experience will be more useful and thus also potentially more important for medical decision making.

Following the ideas of Persson et al.^10^ about balancing the evaluation of and the accessibility to evidence, we also expect the effect to vary by profession. In particular, we expect health care professions with a longer professional history—and consequently a presumably larger body of relevant scientific knowledge on which to draw, however defined—will perceive a larger difference between the two. That is because a practitioner is forced to use whatever evidence is available when making decisions. We conjecture that if the body of scientific information is smaller, other types of information have to be relied upon, which will ultimately lead practitioners to view them as more important but also as being more similar to the scientific evidence (see also Persson et al.^10^).

The length of professional history is difficult to quantify, but we use the year that a profession first began issuing licenses in Sweden as a proxy for the profession’s age: in Sweden. That year was approximately 1733 for physicians, approximately 1833 for dentists, 1958 for nurses, 1991 for dental hygienists, and 1999 for OTs (Swedish Board of Health and Welfare and Act 2010/659^17^). In a previous study,^10^ a similar hypothesis relating the history of a profession and the epistemological beliefs of its members was tested for the concept of proven experience. In that study, the perceived importance of the concept varied with the age of the profession, so that the relatively newer professions perceived proven experience to be closer to equally important to science than professions with a longer history (and arguably more accumulated scientific knowledge). It is, as yet, unknown if the same applies to personal experience.

## Methods

### Study Design

This study uses data from a survey administered to Swedish physicians, nurses, OTs, dentists, and dental hygienists. (Swedish dental hygienists have 3 y of training and are licensed by the Swedish Board of Health and Welfare.) The survey was administered by Statistics Sweden (the nation’s federal statistical agency) to simple random samples of each profession. A first draft of the survey was pretested on a small number of physicians, nurses, and OTs to ensure that questions were intelligible. Surveys were administered in 2 waves. Physicians, nurses, and OTs received their survey in 2018 and dentists and dental hygienists in 2019. Sampling frames were the most recent occupational registries. Seven hundred each of physicians, nurses, and OTs were drawn from the 2015 registry, with sampling frames of 33,618, 91,174, and 7,412 individuals, respectively. Seven hundred dentists and 700 dental hygienists were drawn from the 2017 registry, with a sampling frame of 6,155 dentists and 3,699 dental hygienists.

Participants were not compensated. Research ethics approval was provided by Lund University Regional Ethics Board (decision dnr 2017/428). A preregistration for this study is available at the Open Science Framework (https://osf.io/uwr2c).

### Survey Setting

#### Physicians, nurses, and OTs

Participants were contacted through the post. The first mailing contained information about the survey and a log-in to a Web-based version of the survey (January 2018). The second contained a paper-based version of the survey. The third and fourth contacts were reminders to respond to the survey, in which the last (February 2018) contained yet another paper survey. Data collection closed in April 2018. In total, 47% chose to respond to the Web-based version of the survey, and subsequently 53% responded to the paper version (see Results and Appendix for detailed information about response rates).

#### Dentists and dental hygienists

Participants were contacted through the post in the same pattern as above but with the first contact October 2019 and the last in November 2020. The collection of data closed in January 2020. In total, 59% chose to respond to the Web-based version of the survey (see Results and Appendix for more detailed information about response rates).

### Variables

Participants were asked about the roles and characteristics of science, personal experience, and proven experience in their professions, along with questions about their demographic characteristics and professional experience. Here, we focus on personal experience and science. We used the phrase “personal experience” to make it clear that the experience referred to by that phrase is linked to the individual decision maker, differentiating it from the more collective (and distinctively Scandinavian) meaning of “proven experience” (see above, and the Discussion). A copy of the survey (translated into English) is available in the Appendix.

This study focuses on responses to 3 questions:

1. *“How important is each kind of knowledge for good decisions in health care?”* Responses were given on a 5-point, numbered Likert scale with *not at all important* and *very important* as endpoints. Ratings were made for each of “personal experience,”“proven experience,” and “science.” For dentists and dental hygienists, the question was instead phrased, *“How important is each kind of knowledge for good decisions in dental care?”*
2. *“How certain is each kind of knowledge in health care [dental care]?”* Responses were given on a 5-point, numbered Likert scale with *not at all certain* and *very certain* as endpoints.
3. *“How systematic is each kind of knowledge in health care [dental care]?”* Responses were given on a 5-point, numbered Likert scale with *not at all systematic* and *very systematic* as endpoints.

We operationalize participants’ years of experience in their respective profession by using the amount of time that had elapsed since their professional licensure.

### Quantitative Variables

Our outcome variables represent the difference in ratings between science and personal experience on 3 dimensions: importance, certainty, and systematicity. That leads to 3 variables, 1 for each of questions #1 to #3 above. When participants did not provide a response or provided an uninterpretable response (e.g., selecting multiple categories), we used the modal rating for their profession as an imputed value. We excluded participants if data were missing or uninterpretable for both the rating of science and personal experience.

### Statistical Methods

Our results are based on prespecified linear regression models for the conditional mean of an outcome variable, where the outcome variables are those defined in the previous paragraph. The model includes an interaction with experience, to investigate how the relationships between the outcome variables and profession change as a function of experience:

$$outcome\sim \alpha +\vec{\beta }\cdot (profession\times experience).$$

*Equation 1: Modeling the outcome variables as a function of the professions and experience*.

We chose physicians as the reference category for the variable *profession* and the lowest level of the ordinal variable *experience* to have a value of 0, so that the intercept (alpha) represents the modeled mean value of the outcome variables (*outcome*) for physicians with the least amount of experience. The vector beta contains all of the coefficients for the profession-experience interaction. Model parameters were estimated using R v4.0.3.^22^

## Results

### Participants

Of the 700 licensed members of each health care profession contacted (for a total of 3,500 possible participants), responses were received from 307 physicians, 305 nurses, 368 OTs, 342 dentists, and 305 dental hygienists, leading to a total of 1,627 responses and a response rate of 46%. Of the 1,627 responses, 26 had discrepancies between their self-reported profession and assigned profession that could not be resolved by the authors and were removed from the data set (e.g., participants classified as “physicians” who were medical researchers without a license to practice medicine; see the Appendix for details about each removal). An additional participant was removed because no questions used for the outcome and independent variables were answered. That left a sample of 1,600: 295 physicians, 300 nurses, 365 OTs, 339 dentists, and 301 hygienists. In that sample, 167 uninterpretable or empty values requiring imputation occurred, representing ∼2% of the data used in the analysis.

For comparison, some recent (but shorter) surveys of Swedish physicians and nurses regarding attitudes to assisted suicide, substitution of antiseizure drugs, and parental presence during anesthesia had response rates of 59%, 56%, and 63%, respectively (e.g., Lynøe et al.^23^; Olsson et al.^24^; Andersson et al.^25^).

### Years of Experience

The physician sample was close to bimodal, with physicians having less than 5 y of experience and more than 30 y of experience being most represented. The distribution of experience for nurses and dentists was close to uniform, with the 30+ y of experience category the mode for both. OTs had an almost symmetric distribution around their 16- to 20-y modal category. Hygienists had a nearly uniform distribution of experience. A contingency table of years of experience by profession can be found in the Appendix.

### Outcome Data

**Figure 1:**
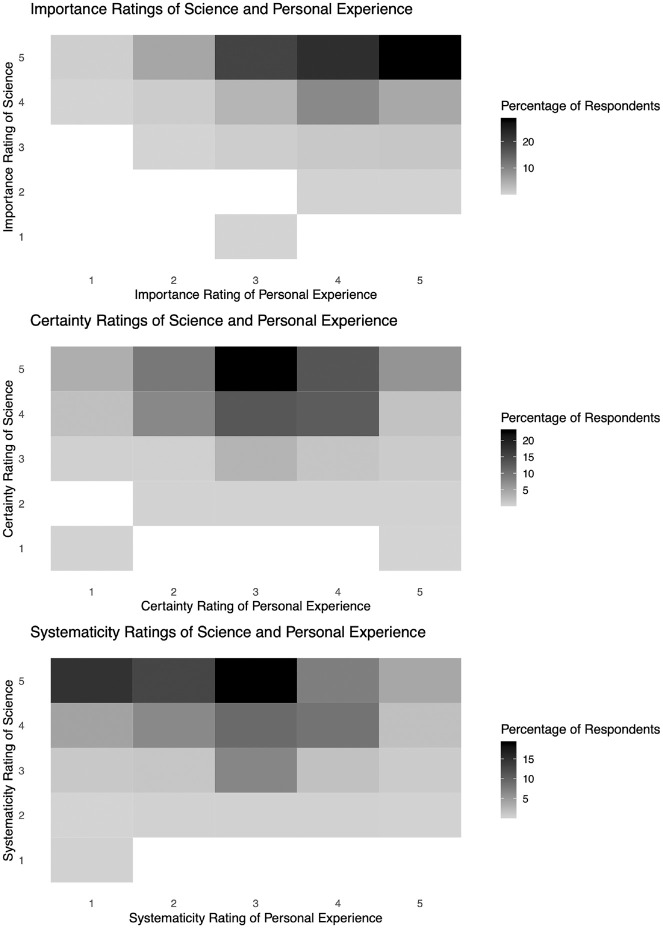
Heat maps showing the joint distributions of ratings of science and personal experience for each of importance, certainty, and systematicity.

Table 1 shows a contingency table for each of the possible values of the 3 outcome variables, separated by profession. Each variable’s possible range is −4 to 4, as each is formed from the difference of 2 Likert-scale variables with a range of 1 to 5. The difference is the rating of personal experience minus the rating for science. Positive values therefore indicate that personal experience received a higher rating on the characteristic, and negative values indicate that science received a higher rating. Table 1 shows that the vast majority of participants rated science more highly than personal experience in terms of their respective importance for decision making, their certainty, and their systematicity, no matter their profession or years of experience. In addition, Figure 1 shows heat maps of the ratings of personal experience and science, showing, for example, that most of the 0s in Table 1 arise from rating both types of knowledge as important. (The Appendix includes a version of Figure 1 for each profession.)

**Table 1:** Outcome Variables (Likert-Scale Ratings of Personal Experience Minus Ratings of Science) on 3 Attributes: (A) Their Importance for Decision Making, (B) Their Certainty, and (C) Their Systematicity^a^

(A)										
Importance	Physician (*n* = 295)		Nurse (*n* = 300)		OT (*n* = 365)		Dentist (*n* = 339)		Hygienist (*n* = 301)	
−4	3	1%	3	1%	2	1%	2	1%	1	0%
−3	29	10%	18	6%	13	4%	21	6%	12	4%
−2	62	21%	56	19%	85	23%	70	21%	46	15%
−1	94	32%	76	25%	86	24%	80	24%	80	27%
0	94	32%	118	39%	135	37%	138	41%	136	45%
1	10	3%	21	7%	34	9%	24	7%	19	6%
2	3	1%	7	2%	9	2%	4	1%	7	2%
3	0	0%	1	0%	1	0%	0	0%	0	0%
4	0	0%	0	0%	0	0%	0	0%	0	0%
(B)										
Certainty	Physician (*n* = 295)		Nurse (*n* = 300)		OT (*n* = 365)		Dentist (*n* = 339)		Hygienist (*n* = 301)	
−4	10	3%	9	3%	14	4%	20	6%	5	2%
−3	35	12%	32	11%	40	11%	45	13%	30	10%
−2	113	38%	90	30%	114	31%	95	28%	85	28%
−1	82	28%	81	27%	115	32%	72	21%	79	26%
0	46	16%	69	23%	69	19%	94	28%	82	27%
1	6	2%	16	5%	9	2%	9	3%	14	5%
2	2	1%	3	1%	4	1%	3	1%	5	2%
3	1	0%	0	0%	0	0%	0	0%	1	0%
4	0	0%	0	0%	0	0%	1	0%	0	0%
(C)										
Systematicity	Physician (*n* = 295)		Nurse (*n* = 300)		OT (*n* = 365)		Dentist (*n* = 339)		Hygienist (*n* = 301)	
−4	68	23%	33	11%	57	16%	54	16%	23	8%
−3	78	26%	43	14%	61	17%	64	19%	23	8%
−2	73	25%	78	26%	103	28%	107	32%	66	22%
−1	47	16%	58	19%	77	21%	40	12%	64	21%
0	24	8%	70	23%	51	14%	58	17%	105	35%
1	4	1%	14	5%	13	4%	13	4%	14	5%
2	1	0%	4	1%	2	1%	3	1%	5	2%
3	0	0%	0	0%	1	0%	0	0%	1	0%
4	0	0%	0	0%	0	0%	0	0%	0	0%

a Negative responses indicate the participant rated science more highly than personal experience on the attribute, while positive responses indicate the opposite.

The Appendix includes supplemental descriptive analyses of the proven experience data, for the interested reader. For example, correlation plots show that proven experience is positively correlated with both science and personal experience but that personal experience is largely uncorrelated with science, reinforcing our interpretation (and the intention of the survey design) that by asking about both personal and proven experience in a population in which the latter is a well-known concept, the former would capture the strictly individual aspects of clinical experience. References Persson et al.,^10,18^ Wahlberg and Persson,^19^ Wallin et al.,^20^ and Dewitt et al.^21^ describe studies focusing on the proven experience data of the survey.

### Main Results

**Figure 2:**
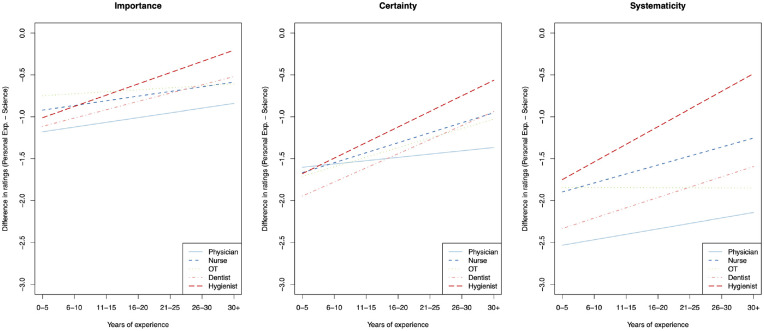
Lines of best fit showing, for each profession, the mean differences in ratings of science and of experience in terms of their importance for medical decision making, certainty, and systematicity. Negative values indicate science received a higher rating.

Table 2 shows the regression coefficients corresponding to the model in equation 1 for each of the 3 outcome variables, with 95% confidence intervals. Figure 2 shows the lines of best fit corresponding to each model.

**Table 2:** Coefficient Estimates Corresponding to the Regression Model in Equation 1

Regression Results	Dependent Variable		
	Difference in Importance (1)	Difference in Certainty (2)	Difference in Systematicity (3)
Nurse	0.26 (−0.07, 0.58)	−0.07 (−0.41, 0.28)	0.64*** (0.23, 1.04)
OT	0.43*** (0.12, 0.73)	−0.11 (−0.43, 0.22)	0.69*** (0.31, 1.07)
Dentist	0.07 (−0.23, 0.36)	−0.34** (−0.66, −0.03)	0.20 (−0.17, 0.57)
Hygienist	0.17 (−0.15, 0.48)	−0.08 (−0.41, 0.26)	0.78*** (0.39, 1.17)
Experience	0.06* (0.00, 0.11)	0.04 (−0.02, 0.10)	0.07* (−0.01, 0.14)
Nurse × Experience	−0.001 (−0.08, 0.08)	0.08* (−0.01, 0.17)	0.042 (−0.06, 0.15)
OT × Experience	−0.03 (−0.12, 0.06)	0.08 (−0.02, 0.17)	−0.07 (−0.18, 0.04)
Dentist × Experience	0.04 (−0.03, 0.13)	0.13*** (0.05, 0.21)	0.06 (−0.04, 0.15)
Hygienist × Experience	0.08* (−0.01, 0.17)	0.15*** (0.05, 0.24)	0.15*** (0.04, 0.25)
Intercept	−1.18*** (−1.38, −0.98)	−1.60*** (−1.82, −1.39)	−2.53*** (−2.79, −2.28)
Observations	1,600	1,600	1,600
*R* ^2^	0.034	0.063	0.105
Adjusted *R*^2^	0.032	0.057	0.100
Residual Standard Error (*df* = 1,590)	1.113	1.183	1.391
*F* statistic (*df* = 9; 1,590)	6.942***	11.780***	20.646***

* *P* < 0.1; **P < 0.05; ***P < 0.01.

Across all 3 models, regression estimates suggest that the majority view science as having more importance, certainty, and systematicity than personal experience, regardless of profession and years of experience—that is reflected in all the lines in Figure 2 lying below 0. Across all the estimates, years of experience diminishes the size of the gap between assessments of science and personal experience, although that effect varies across the 3 outcome variables and by profession, including some instances in which it seems to make no difference. For example, experience has a small effect for physicians across all 3 outcomes but a large effect for hygienists and a varying effect for other professions, as reflected in the coefficients on the interaction between profession and years of experience (Table 2, rows 5–9) and the varying slopes of the lines in Figure 2. Physicians report the largest difference between science and personal experience across all levels of experience in the relative importance and systematicity of the 2 types of knowledge, which is also observed in the certainty outcome among physicians with more than 15 y of experience. In higher values of experience, hygienists see the smallest differences between the 2 types of knowledge (broken red line), and nurses tend to report smaller differences than dentists (dashed blue above orange dots-and-dashes). A version of the model, treating years of experience as a categorical variable, is available in the Appendix.

## Discussion

We asked random samples of physicians, nurses, OTs, dentists, and dental hygienists to rate science and clinical—in this Swedish survey, “personal”—experience in terms of their importance for medical decision making, the certainty of each kind of knowledge, and the systematicity of each kind of knowledge. Overwhelmingly, the differences between the two were smallest for importance and largest for systematicity. We interpret that result as health care professionals being sensitive to the situation-specific aspects of personal experience (allowing it to play an important complementary role to scientific knowledge, see also Persson et al.^10^) but also being acutely aware of its epistemic disadvantages. Almost all participants, regardless of profession or years of professional experience, rated science more highly across all 3 attributes.

Although experience lacks the epistemic advantages of science, which, in its ideal form, is reliably produced and reproducible, experience clearly plays an important role in all types of health care. In fact, the modal difference between their respective ratings of importance is 0 (see Table 1 and Figure 1). One interpretation is that clinical experience is so relevant that it somehow compensates for lacking the certainty and systematicity of science. The idea that evidence-based medicine integrates individual clinical expertise and the best scientific evidence^1^ has been taken to heart by a wide variety of health care professionals.

Among all health care professions surveyed, physicians indicate the largest difference between science and experience, with science having the higher importance, certainty, and systematicity (Figure 2). When professional experience increases, the differences diminish slightly, but science retains its prominence. Given the limited sample size, we cannot comment on differences between different medical specialties. However, we note that dentists—who arguably rely more uniformly on manual skill than physicians do—consistently perceive a smaller difference between science and experience for importance and systematicity and are more strongly affected by years of experience on assessments of the certainty characteristic. Hypothetically, this might suggest that medical specialties with a stronger reliance on manual skills (surgery being the prime example) would demonstrate a similar pattern.

An additional factor that may drive differences between different health care professions is their ability to follow patients over time. Being able to follow a patient would allow them to build personal experience to a greater extent and also to better evaluate their experiences (e.g., allowing for a higher degree of systematicity and certainty). Given the large variation of experiences within health care professions (compare, for instance, a hospital dentist with a practitioner in a small municipality), it is difficult to make any clear predictions regarding professions here, but in principle, we believe that professionals with more patient continuity would perceive personal experience as both more systematic and certain and, as a consequence, likely also as more important.

We predicted that experience would diminish perceived differences between science and experience for all professions. Except for OTs’ views on systematicity, that expectation bore out, but the effect was weak, with confidence intervals for the effect often including 0. The perceived epistemic advantages of science also dominate for individuals with high amounts of personal experience. Health care professionals are acutely aware of the need for science-based practice.

We also predicted that health care professions with a longer professional history (i.e., physicians and dentists) would perceive larger differences between science and experience than those with a shorter professional history (i.e., dental hygienists and OTs). In line with our predictions, dental hygienists perceive the smallest differences between science and experience of all professions surveyed and are also most strongly affected by increased experience, perceiving smaller differences between the two with more years in the profession. OTs are more difficult to evaluate. When it comes to whether science or experience is more important, they are among the professions that perceive the smallest differences, but this is not the case for certainty or systematicity. OTs’ views of the relative merits of science and experience are also the least affected by experience. Given that OTs and dental hygienists have a similarly long professional history (as we have operationalized it) and education of similar lengths, the difference between the two with respect to the epistemic aspects of science (i.e., systematicity and certainty) is hard to explain. That may indicate our operationalization of professional age was too simplistic and overlooks important differences in the types of science available in each profession.

Nurses and physicians appear to have similar views on the relationships between science and experience, although nurses perceive a smaller difference between the two. That is what we expected given the shorter time span in which nursing has been seen as a profession in Sweden. Given the rapid development of nursing science, it would be interesting to return to the differences between physicians and nurses in the future. In our survey, nurses with fewer years in the profession and hence (likely) a more recent education are very similar to physicians, whereas older and more experienced nurses perceive a smaller difference between the certainty of science and experience. At this point, we cannot say if the advances of nursing science or the stronger impact of experience on nurses than on physicians better explain this difference. A similar story can be told about the professional pair of dentist and dental hygienists, with a particularly large difference in the perceived systematicity of science and personal experience, respectively. We conjecture that the slightly more repetitive work of dental hygienists allows personal experience to become more systematic.

### Limitations

None of the professions were sampled in such a way as to ensure a variety or balance of their respective specialties. Although each profession has a standardized initial degree, some, such as medicine, have a wide range of specialties and subspecialties with varied knowledge bases. Our results provide a general overview of epistemic beliefs and are useful for training students in the health care professions before specialization; however, we do not have data to determine specialty-specific results and would expect differences between (say) a sample of psychiatrists and one of orthopedic surgeons.

Furthermore, we instantiate years in the profession as time since licensure. It is impossible to uncouple that from changes in educational systems. For example, it could be that how evidence-based medicine is taught, or the advent of problem-based learning, competency-based assessment, and other pedagogical techniques, have caused the differences we see, rather than years in the profession per se.

## Conclusion

Health care professionals of all kinds base their clinical practice on both the scientific foundations of their profession and their accumulated experience treating patients. In this study, we found that, across 5 health care professions, participants rated science more highly than experience in terms of 3 epistemological characteristics: certainty, systematicity, and importance for decision making. Within each profession, more years in the profession tended to decrease the differences in those ratings, although the effect was small.

Many of the largest differences between professions are seen in terms of systematicity; in particular, the physician-nurse and dentist-hygienist pairs, each of which frequently works together, vary the most on that characteristic. Nevertheless, health care professions appear to share some attitudes toward professional knowledge, despite the variation in both the age of the professions and their accumulated scientific knowledge base. Clinicians of all kinds interact with colleagues in other health care professions, and how the variation (or lack thereof) in their conceptions of knowledge affect policies, training, and ultimately patient outcomes would be a natural research question to pursue next.

## Supplemental Material

sj-docx-1-mdm-10.1177_0272989X241234318 – Supplemental material for Perceptions of Clinical Experience and Scientific Evidence in Medical Decision Making: A Survey of a Stratified Random Sample of Swedish Health Care Professionals Supplemental material, sj-docx-1-mdm-10.1177_0272989X241234318 for Perceptions of Clinical Experience and Scientific Evidence in Medical Decision Making: A Survey of a Stratified Random Sample of Swedish Health Care Professionals by Barry Dewitt, Johannes Persson and Annika Wallin in Medical Decision Making
