# Supplementary material for: Perceptions of Clinical Experience and Scientific Evidence in Medical Decision Making: A Survey of a Stratified Random Sample of Swedish Health Care Professionals
Source: Med Decis Making. 2024 Mar 16;44(3):335–45. doi: 10.1177/0272989X241234318 (PMC10988987; doi:10.1177/0272989X241234318)
Supplement: sj-docx-1-mdm-10.1177_0272989X241234318 – Supplemental material for Perceptions of Clinical Experience and Scientific Evidence in Medical Decision Making: A Survey of a Stratified Random Sample of Swedish Health Care Professionals [file sj-docx-1-mdm-10.1177_0272989X241234318.docx]

**Appendix for *Perceptions of clinical experience and scientific evidence in medical decision making: A survey of a stratified random sample of Swedish healthcare professional***

This appendix contains the following information:

- Lines of best fit for the Equation 1 in the main text, estimated treating years-of-experience as a factor variable
- Figure 1 of the main text, separated by profession
- Correlation tables for the ratings of personal experience, proven experience, and science, by profession
- Stacked bar charts showing how often proven experience was rated higher than both of the other types of knowledge
- Information about the survey dissemination
- Reasons for the removal of data when assigned and self-reported profession did not agree
- An English translation of the survey instrument

The Open Science Framework page for this project is <https://osf.io/gxv6h/>.

**Figure A:** The model (Equation 1) in the main text, estimated with years-of-experience treated as a factor (i.e., categorical), rather than as continuous.
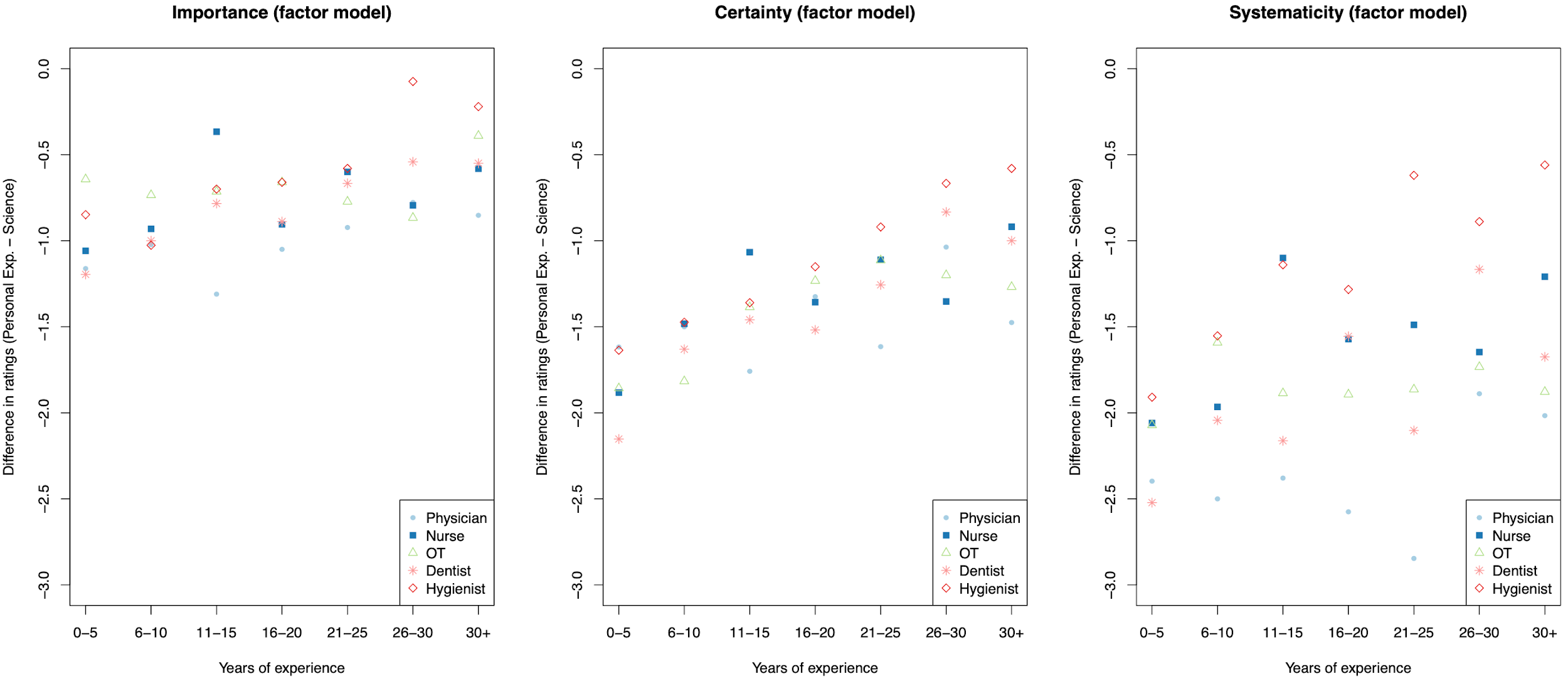


***
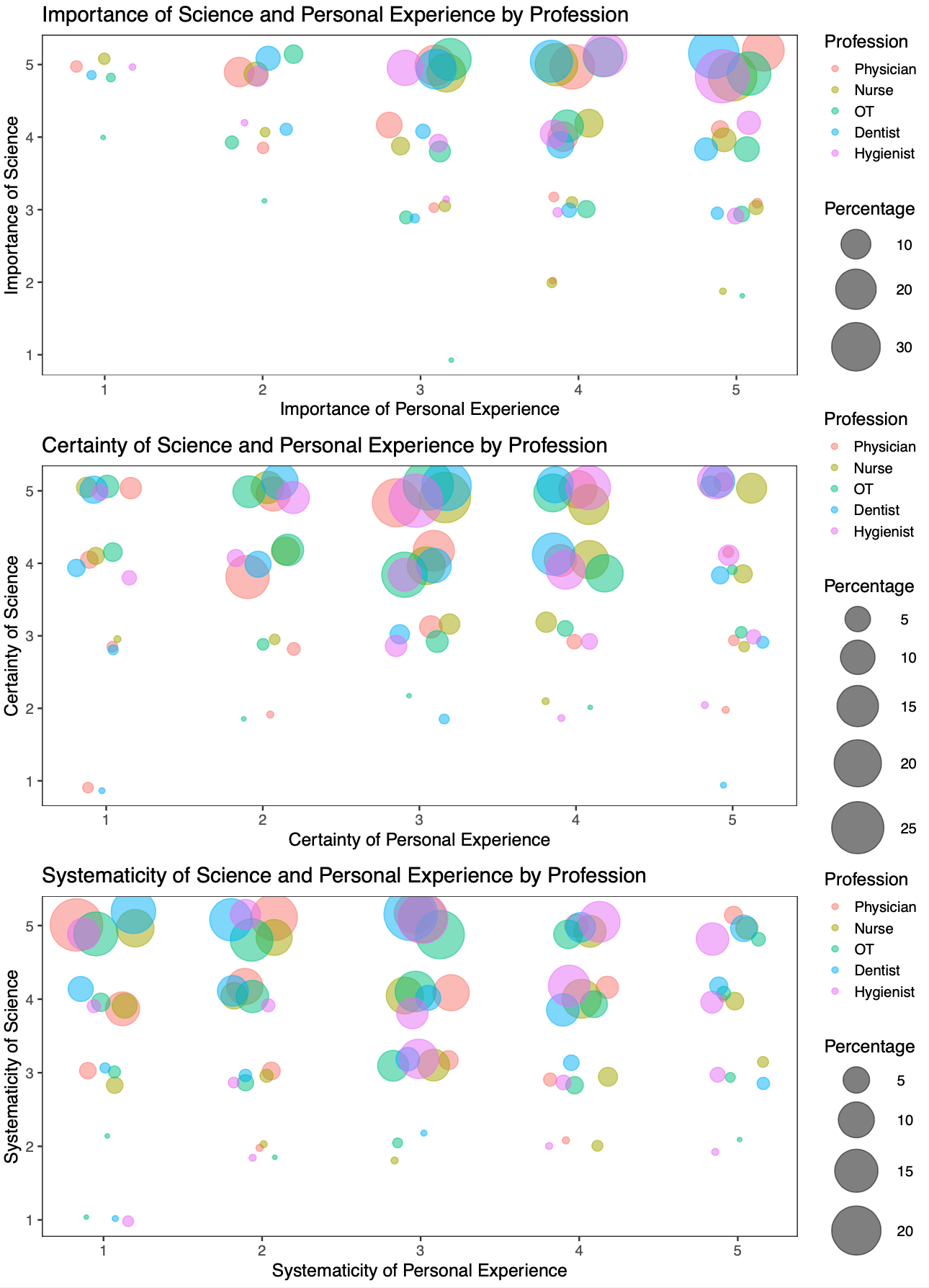
***

**Figure B:** Bubble plots, showing the ratings of science and personal experience for each of importance, certainty, and systematicity, separated by profession. Compare with Figure 1 in the main text.

**
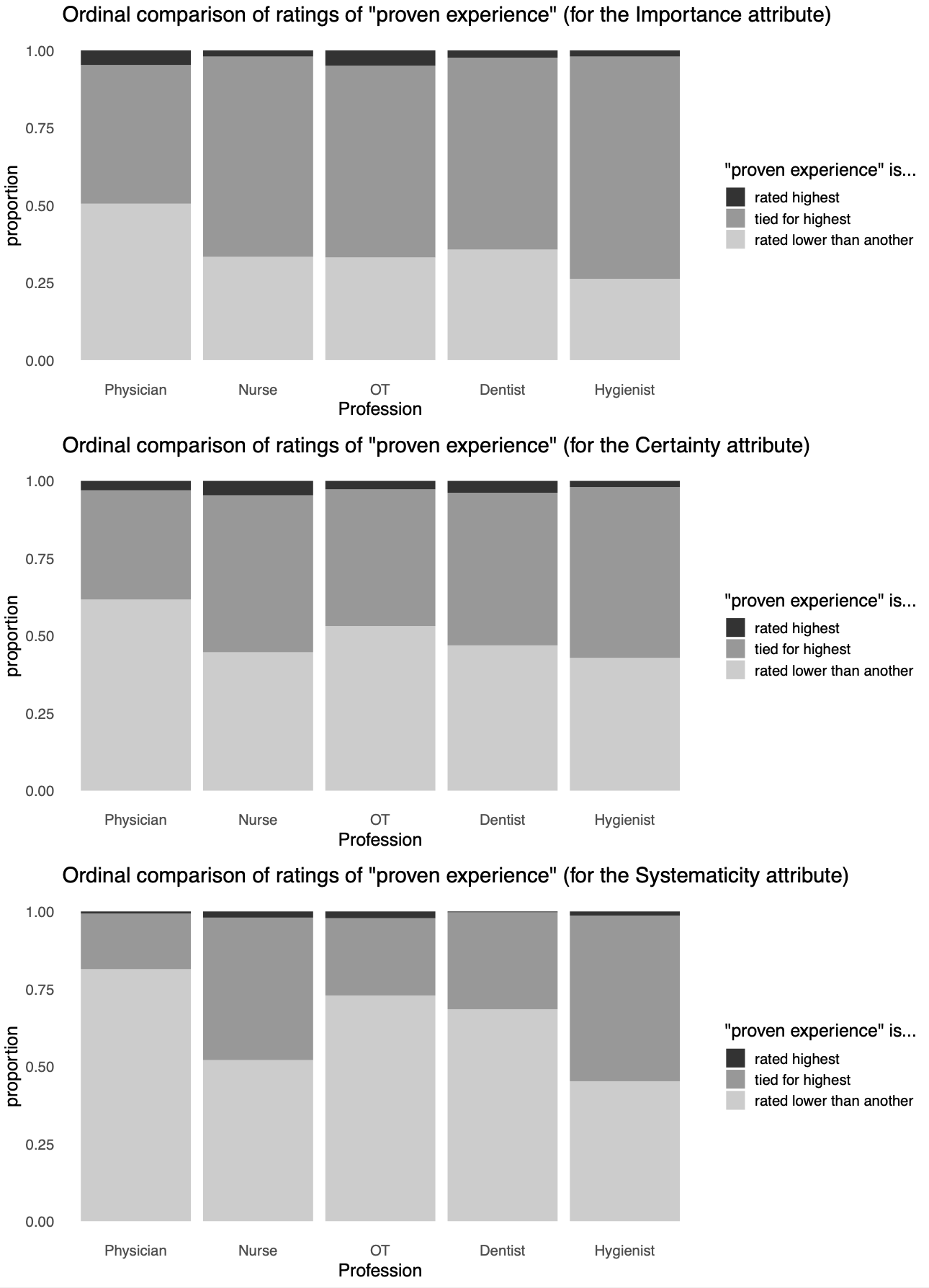
**

**Figure C:** Stacked bar charts showing how proven experience is ranked against the other types of knowledge (i.e., science and personal experience).

***
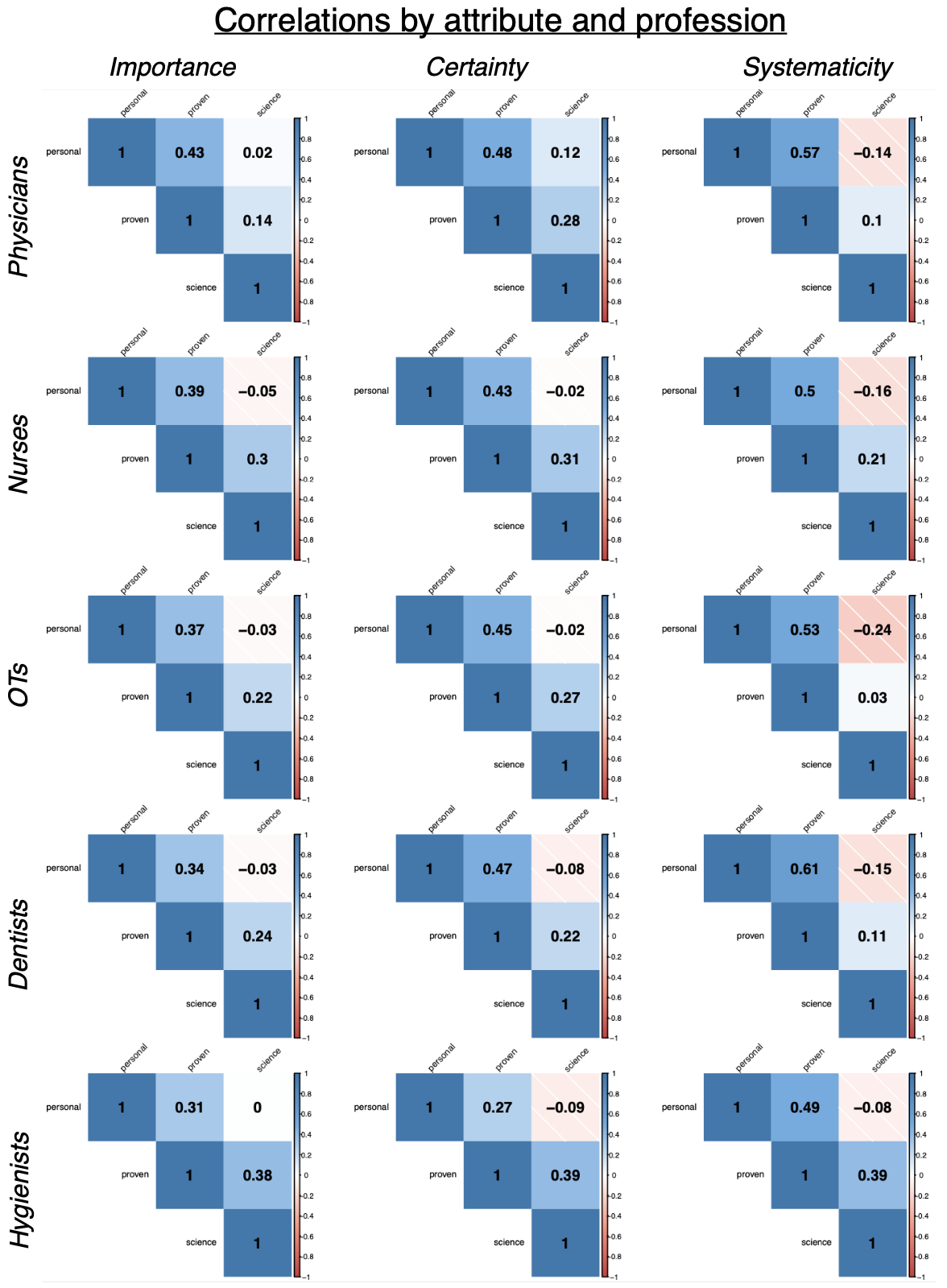
***

**Figure D:** Spearman correlations of responses for each attribute and type of knowledge, separated by profession.

Figure A shows the conditional mean values of the outcome variables, i.e., the fitted values of the model in Equation 1 of the main text with year-of-experience treated as a factor variable (rather than as continuous). It replicates the increasing trend seen in the main model, although there are clearly violations of the monotonicity forced by the linear model in the main text. Of note, nurses in the 11-15 years of experience category give the most similar ratings to personal experience and science across all three attributes, in contrast to the other professions, where those with the most experience tend to provide the most similar ratings. The ratings for systematicity see some of the largest disagreements between the professions (at each level of experience) and within the professions (between levels of experience).

Figure B is a version of Figure 1 in the main text, but with the data separated by profession (displayed using a bubble plot rather than heat map). Note the lower variation of the joint distribution for importance, in particular, compared with systematicity. Note as well that, when science and personal experience receive the same rating, it is often one of being important/certain/systematic.

Figure C shows proven experience ratings compared to those of the other types of knowledge. Proven experience was rarely the strictly highest rated, but often tied for the highest-rated. The main text of the manuscript includes references (10) and (18-21) that focus on proven experience.

Figure D shows Spearman correlations of the attribute ratings for each type of knowledge, separated by profession. Of note, proven experience is often positively correlated with both personal experience and science, but the latter two are uncorrelated or weakly negatively correlated.

***Information about the survey dissemination***

Physicians, nurses and OTs

Number of responses after each survey reminder. Questionnaire closed April 3 2018.

| Date |  | Number of responses |
| --- | --- | --- |
| January 11 2018 | Letter with log in information to web-based survey | 379 |
| January 25 2018 | Letter with physical version of questionnaire | 299 |
| February 2 2018 | Reminder with log in information | 132 |
| February 22 2018 | Reminder with physical version of questionnaire | 170 |

Dentists and dental hygienists.

Number of responses after each survey reminder. Questionnaire closed January 8 2020.

| Date |  | Number of responses |
| --- | --- | --- |
| October 8 2019 | Letter with log in information to web-based survey | 249 |
| October 22 2019 | Letter with physical version of questionnaire | 201 |
| November 6 2019 | Reminder with log in information | 100 |
| November 20 2019 | Reminder with physical version of questionnaire | 97 |

***Reasons for removal of data***

| Reasons for removing participant | Number of participants removed |
| --- | --- |
| Two professions indicated (e.g. stratum nurse, indicates physician certificate) | 10 |
| Missing certificate (e.g. medical researcher but not physician certificate) | 6 |
| Mistake in stratum (e.g. dental nurse not dental hygienist) | 5 |
| Mistake in stratum (e.g. does not work in healthcare) | 5 |

## ***Survey***

Below is an English translation of the survey administered to the study’s participants. The English translation was created by the authors, but all participants took the survey in the original Swedish -- the Swedish version is available by request. The broader aim of the survey was to understand clinicians’ views of their own expertise, and in particular how they understood the legal notions “science and proven experience” governing their work. In this particular paper we target clinicians’ view of the legal role of the notion.

**Proven experience in communication between colleagues**

**A.** First two questions about how well the concept of "proven experience" works in the communication between colleagues.

**1. How certain do you feel that you know what the term "proven experience" means when you use the term?**

| 1 | 2 | 3 | 4 | 5 |
| --- | --- | --- | --- | --- |
| Not at all certain |  |  |  | Completely certain |

If you want to, please feel free to comment on your answer here: [Free text option]

**2. How certain do you think that your colleagues in healthcare generally feel about what the term "proven experience" means when they use the term?**

| 1 | 2 | 3 | 4 | 5 |
| --- | --- | --- | --- | --- |
| Not at all certain |  |  |  | Completely certain |

If you want to, please feel free to comment on your answer here: [Free text option]

**How do you perceive proven experience?**

**B.** In this section of the questionnaire there is a number of statements about proven experience. Please respond based on how you perceive proven experience, regardless of whether it is consistent with how you think others perceive the term.

**3. That there is proven experience of a treatment in the field of health care means that it has been carefully tested in the field of health care.**

| 1 | 2 | 3 | 4 | 5 |
| --- | --- | --- | --- | --- |
| Do not agree at all |  |  |  | Completely agree |

**4. That there is proven experience of a treatment in health care means that it has been shown to be effective in the field of healthcare.**

| 1 | 2 | 3 | 4 | 5 |
| --- | --- | --- | --- | --- |
| Do not agree at all |  |  |  | Completely agree |

**5. That there is proven experience of a treatment in health care means that a group of healthcare professionals together have reached the conclusion that it works.**

| 1 | 2 | 3 | 4 | 5 |
| --- | --- | --- | --- | --- |
| Do not agree at all |  |  |  | Completely agree |

**6. That there is proven experience of a treatment in health care means that it is widely accepted among healthcare professionals.**

| 1 | 2 | 3 | 4 | 5 |
| --- | --- | --- | --- | --- |
| Do not agree at all |  |  |  | Completely agree |

**7. That there is proven experience of a treatment in health care means that its origins lie in the daily activities in health care.**

| 1 | 2 | 3 | 4 | 5 |
| --- | --- | --- | --- | --- |
| Do not agree at all |  |  |  | Completely agree |

**8. That there is proven experience of a treatment in health care means that it does not violate medical ethics.**

| 1 | 2 | 3 | 4 | 5 |
| --- | --- | --- | --- | --- |
| Do not agree at all |  |  |  | Completely agree |

**9. That there is proven experience of a treatment in health care means that it has been used by many health care professionals.**

| 1 | 2 | 3 | 4 | 5 |
| --- | --- | --- | --- | --- |
| Do not agree at all |  |  |  | Completely agree |

**10. Healthcare professionals can have proven experience of carrying out a medical measure.**

| 1 | 2 | 3 | 4 | 5 |
| --- | --- | --- | --- | --- |
| Do not agree at all |  |  |  | Completely agree |

**11. That there is proven experience of a treatment in health care means that it works in the day-to-day activities in health care.**

| 1 | 2 | 3 | 4 | 5 |
| --- | --- | --- | --- | --- |
| Do not agree at all |  |  |  | Completely agree |

**12. That there is proven experience of a treatment in health care means that it is used by healthcare professionals for the current purpose.**

| 1 | 2 | 3 | 4 | 5 |
| --- | --- | --- | --- | --- |
| Do not agree at all |  |  |  | Completely agree |

**13. Proven experience is obvious to anyone who has a lot of experience in the profession.**

| 1 | 2 | 3 | 4 | 5 |
| --- | --- | --- | --- | --- |
| Do not agree at all |  |  |  | Completely agree |

**14. That there is proven experience in treatment means that it is based on the professional’s common sense.**

| 1 | 2 | 3 | 4 | 5 |
| --- | --- | --- | --- | --- |
| Do not agree at all |  |  |  | Completely agree |

**15. Proven experience in health care includes experience of what patients prefer.**

| 1 | 2 | 3 | 4 | 5 |
| --- | --- | --- | --- | --- |
| Do not agree at all |  |  |  | Completely agree |

**16. That there is proven experience of a treatment in health care means that it is used by successful medical units.**

12345

Do not agree at all – Completely agree

**17. That there is proven experience of a treatment in health care means that there is information documented about what has happened when it has been used.**

| 1 | 2 | 3 | 4 | 5 |
| --- | --- | --- | --- | --- |
| Do not agree at all |  |  |  | Completely agree |

**18. That there is proven experience of a treatment in health care means that it has been used in health care for a long time.**

| 1 | 2 | 3 | 4 | 5 |
| --- | --- | --- | --- | --- |
| Do not agree at all |  |  |  | Completely agree |

**Proven experience and other types of evidence**

**C.** In this part of the survey, there is a number of claims about how proven experience relates to other knowledge and evidence in health care.

**19.How important are each of the following types of knowledge for sound decision making in the healthcare sector?**

**Personal experience**

| 1 | 2 | 3 | 4 | 5 |
| --- | --- | --- | --- | --- |
| Not at all important |  |  |  | Very  important |

**Proven experience**

| 1 | 2 | 3 | 4 | 5 |
| --- | --- | --- | --- | --- |
| Not at all important |  |  |  | Very  important |

**Scientific evidence**

| 1 | 2 | 3 | 4 | 5 |
| --- | --- | --- | --- | --- |
| Not at all important |  |  |  | Very  important |

**20. How certain are each of the following types of knowledge in healthcare?**

**Personal experience**

| 1 | 2 | 3 | 4 | 5 |
| --- | --- | --- | --- | --- |
| Not at all certain |  |  |  | Completely certain |

**Proven experience**

| 1 | 2 | 3 | 4 | 5 |
| --- | --- | --- | --- | --- |
| Not at all certain |  |  |  | Completely certain |

**Scientific evidence**

| 1 | 2 | 3 | 4 | 5 |
| --- | --- | --- | --- | --- |
| Not at all certain |  |  |  | Completely certain |

**21. How systematic are each of the following types of knowledge in the healthcare system?**

**Personal experience**

| 1 | 2 | 3 | 4 | 5 |
| --- | --- | --- | --- | --- |
| Not at all systematic |  |  |  | Very systematic |

**Proven experience**

| 1 | 2 | 3 | 4 | 5 |
| --- | --- | --- | --- | --- |
| Not at all systematic |  |  |  | Very systematic |

**Scientific evidence**

| 1 | 2 | 3 | 4 | 5 |
| --- | --- | --- | --- | --- |
| Not at all systematic |  |  |  | Very systematic |

**The legal requirement of science and proven experience**

**D.** In this section of the survey, a few questions follow on how you perceive the requirement of science and proven experience in the legal regulation of healthcare.

According to the Patient Act, patients shall receive healthcare that is in accordance with science and proven experience. The Patient Safety Act states that healthcare professionals have a personal responsibility to carry out their work in accordance with science and proven experience.

**22. How certain do you feel that you know what the term "science and proven experience" means in the legal regulation of healthcare?**

| 1 | 2 | 3 | 4 | 5 |
| --- | --- | --- | --- | --- |
| Not at all certain |  |  |  | Completely certain |

**23. How do you feel about "science and proven experience" being used as quality requirement in the legal regulation of healthcare?**

| 1 | 2 | 3 | 4 | 5 |
| --- | --- | --- | --- | --- |
| Not at all satisfied |  |  |  | Very satisfied |

If you want to, please feel free to comment on your answer here: [Free text option]

**24. How do you feel about "proven experience" being used as quality requirement in the legal regulation of healthcare?**

| 1 | 2 | 3 | 4 | 5 |
| --- | --- | --- | --- | --- |
| Not at all satisfied |  |  |  | Very satisfied |

If you want to, please feel free to comment on your answer here: [Free text option]

**25. Who should - in your opinion - determine how the term "science and proven experience" should be interpreted in the legal regulation of healthcare?**

*You can choose more than one option if you want.*

Doctors and nurses and others in the healthcare professions

Courts / lawyers

SBU/Socialstyrelsen/IVO

Those who health care related research

Don’t know

If something else, please describe it here: [Free text option]

**Some questions about you**

**E.** At last,some questions about who you are.

**26. How old are you**

25 years or younger

26-30

31-35

36-40

41-45

46-50

51-55

56-60

61-65

66 years or older

**27. I am**

Woman

Man

I cannot / do not want to use the options above

**28. What are you currently employed as?**

Nurse

Doctor

Occupational therapist

If something else, please specify: [Free text option]

**29. If you have any specialization, which one is it?**

[Free text]

**30. How long have you had this employment?**

0-5 years

6-10 years

11-15 years

16-20 years

21-25 years

26-30 years

More than 30 years

**31. How long is it since you got your certification**

0-5 years

6-10 years

11-15 years

16-20 years

21-25 years

26-30 years

More than 30 years

**32. Where did you get your license?**

Göteborg

Köpenhamn

Lund

Stockholm

Umeå

Uppsala

Växjö

If something else, please specify: [Free text option]

**33. What academic degree do you have?**

[Free text option]
